# Shedding Light on the Interaction Between Rif1 and Telomeres in Ovarian Cancer

**DOI:** 10.14336/AD.2023.0716

**Published:** 2024-04-01

**Authors:** Paweł Kordowitzki, Szymon Graczyk, Sylvia Mechsner, Jalid Sehouli

**Affiliations:** ^1^Department of Preclinical and Basic Sciences, Faculty of Biological and Veterinary Sciences, Nicolaus Copernicus University, Torun, Poland.; ^2^Department of Gynecology including Center of oncological surgery (CVK) and Department of Gynaecology (CBF), European Competence Center for Ovarian Cancer, Charite, Berlin, Germany.

**Keywords:** ovarian cancer, telomere, TERT, TERC, RIF1, cancerogenesis

## Abstract

Ovarian cancer, more precisely high-grade serous ovarian cancer, is one of the most lethal age-independent gynecologic malignancies in women worldwide, regardless of age. There is mounting evidence that there is a link between telomeres and the RIF1 protein and the proliferation of cancer cells. Telomeres are hexameric (TTAGGG) tandem repeats at the tip of chromosomes that shorten as somatic cells divide, limiting cell proliferation and serving as an important barrier in preventing cancer. RIF1 (Replication Time Regulation Factor 1) plays, among other factors, an important role in the regulation of telomere length. Interestingly, RIF1 appears to influence the DNA double-strand break (DSB) repair pathway. However, detailed knowledge regarding the interplay between RIF1 and telomeres and their degree of engagement in epithelial ovarian cancer (EOC) is still elusive, despite the fact that such knowledge could be of relevance in clinical practice to find novel biomarkers. In this review, we provide an update of recent literature to elucidate the relation between telomere biology and the RIF1 protein during the development of ovarian cancer in women.

## Introduction

**Figure 1.**
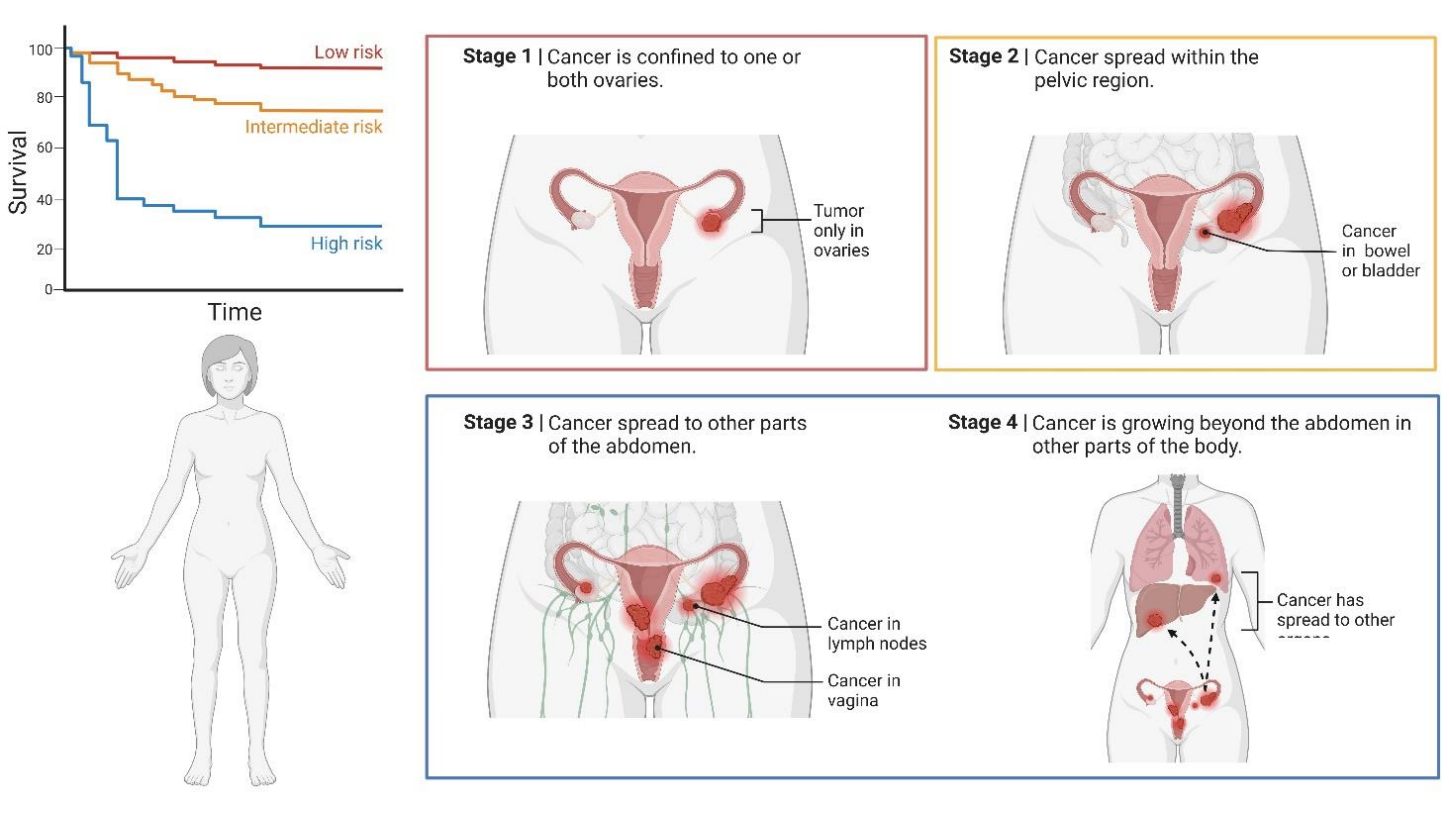
Scheme showing the different stages of ovarian cancer and their characteristics with regard to localisation and expansion in regard to their potential chance of survival.

Owing to its prevalence, neoplasia of the ovary is one of the most common gynecologic, age-independent pathologies in women worldwide [1]. More precisely, a large number of women suffer from epithelial ovarian cancer (EOC), which is estimated to have the lowest survival rate [2]. Although the search for new therapeutic strategies has yielded reliable results, the overall prognosis and survival rate for patients with EOC remains poor [3]. The survival rate of women with ovarian tumors depends, among other things, on the stage of the tumor (Fig. 1), i.e., the higher the stage, the lower the chance of survival. Therefore, elucidating the molecular mechanisms of EOC tumorigenesis and progression is a prerequisite for exploring new therapeutic targets and treating the occurrence of ovarian cancer in women. In this context, telomeres and the RIF1 protein could be very important players. Telomeres are hexameric (TTAGGG) tandem repeats at the tip of chromosomes that shorten as somatic cells divide, limiting cell proliferation, and serving as an important barrier in preventing cancer [4]. Moreover, there is the shelterin complex at the telomeric region and the enzyme telomerase, which consists of two components, the RNA template (TERC), and the catalytic subunit, the telomerase reverse transcriptase (TERT) [5]. Among other species, the human TERT (hTERT) is responsible for telomerase activity, and therefore, for transcription, too. Interestingly, hTERT is upregulated in most ovarian tumors [6] and has been shown to be a central regulator of many cancer traits, like proliferation, survival, and characteristics of cancer stem cells [7-9]. Noteworthy, EOC stem cells do reflect these latter-mentioned properties, too [10-12]. Although hTERT appears to be a reliable biomarker and therapeutic target for EOCs, its relevance in neoplastic cells remains elusive. Numerous cancer types show typical maintenance of telomere length in their cells since among other mechanisms the catalytic subunit is responsible for adding the hexameric sequence [13]. It is interesting though that the Replication Time Regulation Factor 1 (RIF1) binds directly to the promoter for hTERT enabling its expression, and in consequence, it plays an essential role in the regulation of telomere length [14]. Besides, RIF1 impacts the selection of DNA double-strand break (DSB) repair pathway and the regulation of replication timing [15-18], and is highly expressed in mouse embryonic stem cells, too [19-21]. Upregulation of RIF1 in breast cancer tissues has been reported and the knockdown of RIF1 reduced cell growth and increased susceptibility of uterine cervical cancer cells to cisplatin [22-23]. However, the specific role of RIF1 in EOC needs further elucidation.

In this review, we aim to shed light on the importance of the relationship between telomere biology and the RIF1 protein during cancerogenesis, especially during the development of ovarian cancer in women. Moreover, we intend to answer the question of whether TERT or RIF1 could be established as new biomarkers for the early detection of ovarian cancer regardless of the patient’s age.

## The relationship between Rif1 and telomeres in ovarian cancer

Telomere shortening reflects a so-called mitotic clock in somatic cells since by every cell division and with advancing age, the chromosome’s tip gets shorter and therefore not only determines the life span of somatic cells but also serves as an intrinsic barrier for oncogenic transformation [24]. The latter-mentioned physiologic event is enabled due to specific pathways, such as the amplification of the gene encoding for TERT [25], the influence on transcriptional activators of TERT [26], and the cytosine methylation at CpG islands close to the TERT promoter [27]. Telomere length in specific cancer can be maintained via a telomerase-independent pathway, meaning an alternative elongation of telomeres [28], based on homologous recombination [29]. As previously mentioned, an elevated expression of hTERT and therefore, its positive effect on telomere length is crucial for the process of tumour development. Previous investigations provided evidence that a plausible reason for this elevated transcription is mutations at the promoter of hTERT [30, 31]. In the study of Wu and co-workers [30] the before-mentioned mutations were described in 15.9% of patients suffering from clear cell carcinomas of the ovary. Worth mentioning that the mutation of the hTERT promoter seems not to be present at the beginning of oncogenesis and appears to be linked with the lack or downregulation of ARID1A, a tumour suppressor gene, in ovarian clear cell carcinomas (OCC) [30]. Previous research provided evidence that the mutations are positioned at two loci [32,33]. Based on other studies and the study of Wu et al. [30] it appears that the pathogenetic mechanisms between OCC and ovarian endometrioid carcinoma are divergent. Other studies have also confirmed among others the mutation of the gene encoding for ARID1A mutation as an property during the early onset of OCC development but does also occur in endometriotic cysts [34,35]. Additionally, genome-wide analysis has also confirmed that ARID1A mutations have been detected in ovarian clear cell carcinomas [36,37]. According to the so-called telomere crisis theory, which is relevant for tumourigenesis, the accumulated telomere attritions cause senescence and/or harmful genomic instability [38].

As a logical consequence, precancer cells aim to escape from the latter-mentioned telomere crisis to survive, and during the phase of telomere crisis, only those cells with adequate maintenance of telomere length will progress due to the selection pressure [30]. However, the data about TERT promoter mutations in the gynecological tract are rare [39,40]. Interestingly, it has been revealed that in patients diagnosed with OCC (FIGO stages I and II), mutation of the TERT promoter appeared to be an independent prognostic factor in combination with significantly shorter overall survival. Besides, in patients with recurrent OCC (early FIGO stage), mutation of the TERT promoter was significantly correlated with a relapse within six months [41].

**Figure 2.**
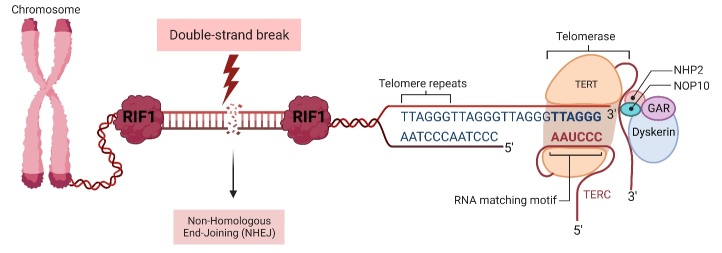
**Scheme showing directed Rif1-DNA interactions required to prevent telomerase and inadvertent activation of checkpoints at chromosome ends**. In the event of a double strand break, the Rif1 seals the broken ends and opens access to tip resection machinery. As a result, the double-strand breaks ends are stabilized, promoting their retrocession by non-homologous end-joining (NHEJ).

## Relevance of RIF1 in cancerogenesis and different cancer types

As previously mentioned, understanding the mechanisms and regulators controlling cancerogenesis has become a priority for specialists worldwide. Given that Rif1 is involved in telomeric regulation (Fig. 2), the search for its role in tumorigenesis has begun. In one study, it was shown that as telomere length decreases, the amount of free Rif1 increases due to the loss of its specific association with Rap1 and thus association with telomere ends. The abnormally increased amount of free Rif1 promotes tumourigenesis due to its impact on genomic instability and rearrangement of the chromosomes [42]. Furthermore, Rif1 may act as an anti-checkpoint shield in repairing defective double-strand breaks (DSB) of DNA in breast cancer, and inhibition of its expression sensitizes cancer cells to drugs [42,43]. A direct mechanism in which Rif1 promotes tumour growth has also been revealed. In human er EOC cells, there is a close interaction between Rif1 and hTERT. As it turns out, Rif1 binds directly to the promoter for hTERT allowing its expression, while Rif1 knockout inhibited ovarian tumour growth [42-44]. Furthermore, Rif1 knockout inhibits EOC cell migration and markers of the epithelial-mesenchymal transition leading to apoptosis and G2 cell cycle arrest of EOC cells [43]. Interestingly, also in EOC, Rif1 gene knockout sensitized its cells to drugs (cisplatin) and also platinum-based chemotherapy through inhibition of NER proteins in cancer cells [44]. A positive correlation between Rif1 and lung carcinoma cells was detected in another study. Importantly, a significant correlation was found between Rif1 and the regulators of proliferation signalling and maintenance of cancer stem cell characteristics, Wnt/β-catenin. As in previous studies, inhibition of Rif1 expression limited tumour growth, while its overexpression promoted tumour growth through activation of the PP1-AXIN trail resulting in induction of Wnt/β-catenin pathways [45]. Research into the function of Rif1 in tumourigenesis has yielded new solutions and alternative treatment pathways for various tumour types. Solutions based on inhibition of the expression of this protein have proven beneficial in the negative regulation of tumour growth through the Rif1-hTERT or Rif1-PP1/AXIN- Wnt/β-catenin interaction pathways, and it is therefore believed that further research into Rif1 in tumorgenesis may provide many effective solutions for cancer therapy [45].

## Crosstalk between Rif1 and telomeres in other cells

Although Rif1 is conserved in cells from yeast to humans, the way it binds in organisms differs by interacting with different binding fractions. In budding yeast cells (Sacharomyces Cervisae - scRif1), the connection of the Rif1 protein with the respective domain on its C-terminal is mediated by Rap1 [46]. A second Rif2 protein is attached to this complement, containing two binding sites on Rap1 which together form a complex involved in telomere end protection. As it turns out, the N-terminal domain of scRif1 allows direct attachment to telomere ends. This connection is made possible by the unique shape of the N-terminal domain which adopts the contour of a shepherd's stick [46,47]. Further studies have shown that this region forms a conserved HEAT domain that is also responsible for dimerisation of scRif1 structures [47]. While Rif1 in budding yeast (Sacharomyces pombe - spRif1) is also involved in telomere-end protection, its mode of binding is somewhat different. Although the rap1 protein has been shown to be present, the main binding protein for the connection between the telomere end and spRif1 is Taz1 [48]. In the methylotrophic yeast Rif1 (Hansenula polymorpha hpRif1), it has also been shown to bind to Rap1, but this is not the main mechanism recruiting this hpRif1. Firstly, the hpRap1 protein has two fractions, hpRap1A and hpRap1B of which the first has no binding sites on telomeres and is associated rather with subtelomeric regions while the latter is responsible for telomere binding to dsDNA [49]. Secondly, the same authors suggest that hpRif1 binding to the hpRap1B domain is mainly responsible for recombination and not telomere length [49]. Subsequent studies have shown that Rif1 interacts with the Cdc13 complex, which in turn obtains an association with Stn1 similar to that in S. Cervisae [50]. Finally, it has been shown that the main hpRif1 binding fraction is the Ku80 heterodimer, which is able to bind telomere ends via Stn1 [51]. Although in mammals, including humans, the presence and ability of Rap1 protein to bind to telomere ends via the TRF1-TRF2 complex has been demonstrated, so its interaction with hRif1 at this site has not been reported [46]. This is primarily related to the distinct function of hRif1, where it is involved in DNA damage repair mechanisms via crosstalk with 53BP1 by protecting non-homologous DNA ends [46]. Furthermore, in mouse Rif1 (muRif1) a compact structure can be formed which is able to bind several DNA G-quadruplexes (G-4 DNA) [52]. In turn, G-4DNA has been shown to be involved in binding to the telomere-binding factor (TRF) in humans [53], therefore, researchers point to a high role for G-4 DNA in telomere end protection [54]. The situation is different when it comes to the genome of cells of the genus Drosophila. The mechanism of telomere-end protection is based on the action of retrotransposons [55], and not telomerase as in most organisms known to date, hence binding complexes such as Rap1, Taz1 or TRF are not present in the telomere-end region. Although the presence of the binding factor Rif1 has been demonstrated, the knowledge of the main location of dRif1 is incompletely understood. Studies indicate that dRif1 is associated with the three most commonly described functions of this protein, namely control of replication time, being its main inhibitor through interaction with protein phosphatase and S-phase kinases [56,57]. Interestingly, studies have shown dRif1 does not localize to telomeres obtained from yeast while the hRif1 homologue is capable of this interaction, suggesting that it retains the ability to bind telomere ends [58]. The above data show that Rif1 orthologues exist from yeast to humans, but their localisation and binding factors are different. In addition, not all Rif1 orthologues retained the ability to bind telomeres which translates into their later functions, so understanding these orthologues turns out to be crucial for understanding the activity and localisation of this regulator.

## Rif1 functions in different species

Since the regulatory protein Rif1 was first discovered in budding yeast cells, interest in its function in relation to telomere end protection has increased significantly. Thanks to the binding factor scRif1, it is possible for it to bind at telomere ends and participate in telomere protection through the Rif1 recruitment factor - Rap1 [13]. The same function is attributed to spRif1 as well as hpRif1 but, as previously mentioned, binding takes place using other linking fractions [48,51]. A study using PAL cells, i.e. model yeast organisms lacking telomerase function and telomerase capacity, showed that the concentration of Rif1 at an appropriate, constant level enabled telomere ends to be protected from spontaneous senescence. Moreover, while Rif1 was overexpressed the initiation of cell ageing and also the accumulation of DNA DSBs occurred [42]. When one of the complexes required for telomere capping is lost, scRif1 takes over as the major regulator of telomere function which only points to its critical role in telomere end protection [59,60]. However, despite its well-defined role in regulating telomere elongation, one study determining the effect of scRif1 telomere gene mutations on telomere origins firing showed that it was not associated with telomere length regulation suggesting that, despite origin gene mutations, the main function of scRif1 was still active [61]. In addition, much attention is focused on the degree of phosphorylation of the SCD domain of scRif1 since, according to the authors, it can positively as well as negatively regulate telomere length. One of the phosphorylation sites is Tel1, which is a homologue of the human ATM kinase. Synthetic telomeres lacking this kinase showed an increase in length, but there was no significant effect on chromosomal telomeres. The authors indicate that mutation of both the Tel1 fraction on the SCD domain and other telomeric DNA binding proteins could be used to study other functions of scRif1 in DNA repair and replication [62] was confirmed by one of the most recent studies analyzing mutation of the Rif1 gene and Tel1 simultaneously [63]. This situation is prevented due to its close crosstalk with protein phosphatase 1(PP1), for the reason that by recruiting PP1 to scRif1, telomerase, in general, Tel1 effectively suppresses telomere end repeat elongation [64]. In addition to telomere end protection, Rif1 has been shown to be intimately involved in the process of non-homologous DNA end joining (NHEJ) taking part in DNA repair [47], which has also been demonstrated in mammalian cells where it is indicated that this is one of the main mechanisms regulated by hRif1, without showing a direct effect on telomere end protection [65]. The accumulation of scRif1 itself at DSB sites is made possible by the S-acylation of the N-terminal domain of scRif1 through the palmityl acyltransferase complex pfa4 [66]. In addition, scRif1 acts as a checkpoint inhibitor by inhibiting DNA damage response. This prevents mutation of certain DNA end-strand genes and excessive telomere shortening, which would lead to cell death [60,67]. Ortholog of Rif1 in another yeast species Candida Glabrata did not exhibit telomere-protective functions while it was involved in subtelomeric DNA silencing [68].

It is also worth focusing on the role of mammalian mRif1 because, as previously mentioned, it does not bind directly on telomeres preventing the positive or negative effects of restriction enzymes. Instead, a very interesting correlation between the ZSCAN4 gene of embryonic stem cells (ECSs) and Rif1 was indicated. The latter-mentioned gene encodes for a specific protein that is responsible for the recombination-dependent telomere elongation mechanism and is required for normal, balanced cell growth [69]. In addition, the recombination-dependent telomere elongation mechanism through ZSCAN4 acts autonomously from the other mechanism regulated by telomerase. This seems to be confirmed by a study conducted on mouse embryonic cells and human ALT tumour cells during telomerase-encoding gene knockout. It was shown that the protein which is mainly involved in the overriding function of telomere homeostasis was encoded by ZSCAN4 [70]. It was indicated that the hRif1 protein may be involved in limiting its overexpression, through a mechanism of subtelomeric silencing of ZSCAN4 thus leading to genomic stability of ECSs [71]. The regulatory mechanism is based on a specific interaction at the promoter of the ZSCAN4 gene combining with histone H3K9 methyltransferases leading to subtelomeric silencing [21,71]. In the same study, knockout of hRif1 was performed in ECSs cells leading to telomere hypercombination, elongation, and heterogeneity. Furthermore, knockout of both the gene encoding hRif1 and ZSCAN4 partially rescued defective embryogenesis by protecting telomere recombination phenotypes [21], thus indicating a key role for hRif1 in an indirect mechanism of telomere protection.

The above data report that Rif1 protein homologues in cells from yeast to humans play a very important role in maintaining genetic stability, being one of the critical regulators of telomere biology. However, the growing interest in this protein over the past few years has led scientists to search for further important functions of Rif1 given its highly conserved nature. Among others, it has been pointed out that it prevents too early activation of the onset of replication [72], DNA double-strand breaks repairs [73], or maintains genomic stability in mouse embryonic cells [74].

## Rif1 and tumor microenvironment in ovarian cancer

**Figure 3.**
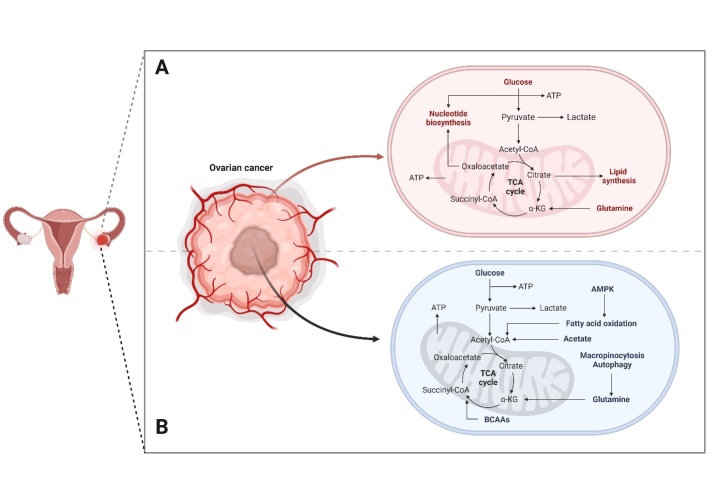
**Scheme showing the cancer cell metabolism in nutrient replete and nutrient deprived conditions**. **(A)** Cancer cell metabolism in nutrient-replete conditions, **(B)** Cancer cell metabolism in nutrient-deprived conditions.

**Figure 4.**
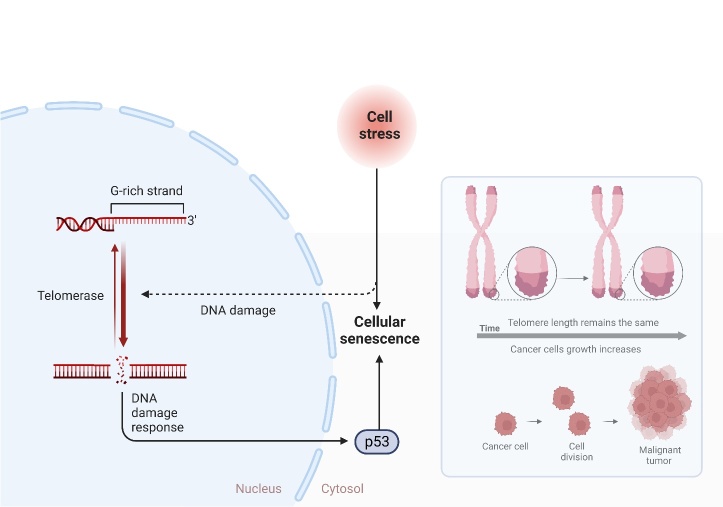
Scheme showing the pathway of cell stress inducing cellular senescence.

The cancer microenvironment is considered to be not only involving the transformation of tumor cells themselves, but also interactions between them and non-cancerous cells, as well as the conditions around the tumor cells. One of the most commonly mentioned facts in this regard is the poor oxygen availability in the tumor micro-environment leading to intense, disorganized angiogenesis within the tumor. This induction depends on a heterodimer formed of hypoxia-inducible factors HIF-α and HIF-β which, under hypoxic conditions, migrate to the cell nucleus to induce VEGF and stimulate angiogenesis [75,76]. As a result of the developing hypoxia, cells reprogram the way they obtain energy from oxidative phosphorylation to anaerobic glycolysis. For this reason, there is an accumulation of lactic acid in the tumor microenvironment causing a drop in pH below 6.8. The acidified environment inhibits the response from the immune system and promotes malignancy and metastasis of tumor cells [77]. In addition, acidic pH influences tumor metabolic remodeling, which in consequence impacts tumor cell growth due to the reorganization of metabolic pathways [78]. Interactions between tumor cells and other cell types have been reported [79], for instance, ovarian cancer cells which program the cellular targets of tumor stroma fibroblasts to maximize glutamine anabolism positively affecting tumor metabolism and infiltrative growth [80]. The lipid chaperone protein FABP4 of ovarian cancer cells enables growth, development and enhances metastasis in a lipid-rich environment, leading to intense proteo-metabolic changes manifested by intensification of lipid metabolism [81]. It seems that, in addition to intercellular interactions, the correlation between tumor cell organelles also exerts an influence on the corresponding adaptation relative to the tumor microenvironment. Thus, as a source of metabolism and energy acquisition, it is the mitochondria of tumor cells and their interactions mainly with the cell nucleus that enable adaptation to various conditions including oxidative or starvation stress [82,83]. The latter arises as a result of a nutrient-deprived condition thus activating modified energy acquisition pathways (Fig. 3), in addition to the natural derivation of pyruvate by glycolysis, including through the activation of adenosine monophosphate-activated protein kinase (AMPK) as a result of a deficiency in intracellular glucose concentration [84], which are then involved in the production of acetyl-coenzyme A. Branched-chain amino acids (BCAAS) are also involved as a result of nutritional deficiencies, which, in addition to being incorporated into the tricarboxylic acid cycle (TCAc), also contribute to glutamine synthesis [80]. Correlations and information exchanges in the tumor microenvironment promote tumor aggression and infiltrative growth, but there are some pathways for cancerogenesis suppression induced by cellular stress. As critical regulators, it considers the p53 factor and p16ink4A which, upon DNA damage, telomere erosion, oncogene hyperactivation and inactivation of onco-suppressors, induce a program of cellular senescence and later cell necrosis to prevent neoplastic transformation (Fig. 4). On the other hand, despite the inhibition of proliferation of these cells, their metabolic activity is still active, and the released biomolecules can induce chronic inflammation and predispose to a pro-cancer microenvironment [85].

The above data show that there are a number of endogenous factors that promote tumor cell aggression and proliferation. The tumor microenvironment, intercellular, and inter-organelle interactions induce mechanisms of tumor survival and malignancy. Therefore, in the context of combating carcinogenesis, targeted therapy on the pathways of inhibition of the above interactions seems to be most beneficial which has already been documented in many studies [86,87], There are also defense pathways on the part of the organism, including through regulators of p53 and p16ink4A, however, it needs to be determined under what conditions cellular senescence produces positive and under what conditions negative effects.

## Conclusions and potential clinical implications

In summary, there are many regulatory proteins that maintain genomic stability. However, some of them are critical for cell survival. Rif1 protein, as a highly conserved protein in cells from yeast to humans, unsurprisingly has very similar functions, but using different mechanisms to achieve its target. Nevertheless, it can be pointed out that Rif1 is an overarching regulator in the cells of many organisms, including humans, preventing the replicative senescence mechanism from occurring too quickly by excessively shortening telomeres. Thus, the telomeric function of the Rif1 protein has found application in cancer therapy with satisfactory results, but further research is needed to fully define the function of this protein in tumorigenesis mechanisms. It is also important to be aware of the other functions of Rif1 and of the series of correlations with various regulators that together maintain genomic stability in the cells of many organisms. For this reason, the Rif1 protein can be considered one of the main factors for cell survival not only humans. In summary, it can be recommended that ovarian cancer patients should be diagnosed for TERT promoter mutations and should be additionally under adequate follow-up care in the months after chemotherapy. With regards to ovarian clear cell carcinomas, the screening for mutations in the genes of ARID1A, PIK3CA, and ZNF217, can be judged as an inadequate prognostic marker [88-91]. However, the detection of mutation in the TERT promoter in early FIGO stages of ovarian cancer patients appears to be a reliable prognostic marker. Importantly though, regular screening for TERT promoter mutations could be used to develop novel therapeutic strategies, to avoid the development of chemoresistance [41]. Nevertheless, there are very limited data about the impact of these observations in the specific subtypes of ovarian cancer, including low and high-grade tumors and the specific histological type such as clear cell cancers. Further investigations should also focus on the impact of Rif1 and telomeres on the survival rate and specific ovarian cancer subtypes and different FIGO stages since these are still research gaps that require further elucidation. As it has been shown in a recent study, short-time suppression of TERT reduces cell growth [31]. Based on this finding new antineoplastic compounds could be established which temporarily inhibit TERT. All in all, there is no doubt that both RIF1 and telomeres do play a critical role during the pathogenesis of ovarian cancer in women regardless of age, therefore, they can be used in clinical practice as potential diagnostic and prognostic biomarkers. Our review aimed to shed light on the importance of the latter-mentioned factors, and due to the limited number of studies on the crosstalk and its relevance for ovarian cancer patients, we aimed to stimulate researchers to perform new experiments on this interesting topic.

## References

[1] Zhang Y, Wang Y, Valdivia A, Huang H, Matei D (2023). DOT1 L Regulates Ovarian Cancer Stem Cells by Activating β-catenin Signaling. Mol Cancer Res, 21(2):140-54. 36318113 10.1158/1541-7786.MCR-22-0418 PMC9898143

[2] Chen L, Yao Y, Sun L, Zhou J, Miao M, Luo S, et al (2017). Snail Driving Alternative Splicing of CD44 by ESRP1 Enhances Invasion and Migration in Epithelial Ovarian Cancer. Cell Physiol Biochem, 43(6):2489-504. 29131012 10.1159/000484458

[3] Xie X, Yang M, Ding Y, Yu L, Chen J (2017). Formyl peptide receptor 2 expression predicts poor prognosis and promotes invasion and metastasis in epithelial ovarian cancer. Oncol Rep, 38(6):3297-308. 29039544 10.3892/or.2017.6034 PMC5783575

[4] Zhang N, Zhang R, Zou K, Yu W, Guo W, Gao Y, et al (2017). Keratin 23 promotes telomerase reverse transcriptase expression and human colorectal cancer growth. Cell Death Dis, 8(7):e2961-e2961. 28749462 10.1038/cddis.2017.339 PMC5550880

[5] Blackburn EH (1991). Structure and Function of Telomeres. Nature, 350:569-573. 1708110 10.1038/350569a0

[6] Shay JW, Bacchetti S (1997). A survey of telomerase activity in human cancer. Eur J Cancer, 33(5):787-91. 9282118 10.1016/S0959-8049(97)00062-2

[7] Liu Z, Li Q, Li K, Chen L, Li W, Hou M, et al. (2013). Telomerase reverse transcriptase promotes epithelial-mesenchymal transition and stem cell-like traits in cancer cells. Oncogene, 32(36):4203-13. 23045275 10.1038/onc.2012.441

[8] Low KC, Tergaonkar V (2013). Telomerase: central regulator of all of the hallmarks of cancer. Trends Biochem Sci, 38(9):426-34. 23932019 10.1016/j.tibs.2013.07.001

[9] Xie M, Chen Q, He S, Li B, Hu C (2011). Silencing of the human TERT gene by RNAi inhibits A549 lung adenocarcinoma cell growth in vitro and in vivo. Oncol Rep, 26(4):1019-27. 21743972 10.3892/or.2011.1383

[10] Kubo T, Takigawa N, Osawa M, Harada D, Ninomiya T, Ochi N, et al. (2013). Subpopulation of small-cell lung cancer cells expressing CD 133 and CD 87 show resistance to chemotherapy. Jpn J Cancer Res, 104(1):78-84. 10.1111/cas.12045 PMC7657141 23066953

[11] Bertolini G, Roz L, Perego P, Tortoreto M, Fontanella E, Gatti L, et al. (2009). Highly tumorigenic lung cancer CD133+ cells display stem-like features and are spared by cisplatin treatment. Proc Natl Acad Sci, 106(38):16281-16286. 19805294 10.1073/pnas.0905653106 PMC2741477

[12] Huang BS, Luo QZ, Han Y, Huang D, Tang QP, Wu LX (2017). MiR-223/PAX6 axis regulates glioblastoma stem cell proliferation and the chemo resistance to TMZ via regulating PI3K/Akt pathway. J Cell Biochem, 118(10):3452-3461. 28332226 10.1002/jcb.26003

[13] Harley CB. Telomerase and cancer therapeutics. Nat Rev Cancer. 2008 Mar; 8(3):167-79. doi: 10.1038/nrc2275. 18256617

[14] Hardy CFJ, Sussel L, Shore D (1992). A RAP1-interacting protein involved in transcriptional silencing and telomere length regulation. Genes Dev, 6(5):801-14. 1577274 10.1101/gad.6.5.801

[15] Hayano M, Kanoh Y, Matsumoto S, Renard-Guillet C, Shirahige K, Masai H (2012). Rif1 is a global regulator of timing of replication origin firing in fission yeast. Genes Dev, 26(2):137-150. 22279046 10.1101/gad.178491.111 PMC3273838

[16] Yamazaki S, Ishii A, Kanoh Y, Oda M, Nishito Y, Masai H (2012). Rif1 regulates the replication timing domains on the human genome. EMBO J, 31(18):3667-3677. 22850674 10.1038/emboj.2012.180 PMC3442267

[17] Alver RC, Chadha GS, Gillespie PJ, Blow JJ (2017). Reversal of DDK-Mediated MCM Phosphorylation by Rif1-PP1 Regulates Replication Initiation and Replisome Stability Independently of ATR/Chk1. Cell Rep, 18(10):2508-2520. 28273463 10.1016/j.celrep.2017.02.042 PMC5357733

[18] Foti R, Gnan S, Cornacchia D, Dileep V, Bulut-Karslioglu A, Diehl S, et al. (2016). Nuclear Architecture Organized by Rif1 Underpins the Replication-Timing Program. Mol Cell, 61(2):260-73. 26725008 10.1016/j.molcel.2015.12.001 PMC4724237

[19] Adams I, McLaren A (2004). Identification and characterisation of mRif1: A mouse telomere-associated protein highly expressed in germ cells and embryo-derived pluripotent stem cells. Dev Dyn, 229(4):733-744. 15042697 10.1002/dvdy.10471

[20] Wang J, Rao S, Chu J, Shen X, Levasseur DN, Theunissen TW, et al. (2006). A protein interaction network for pluripotency of embryonic stem cells. Nature, 444(7117):364-368. 17093407 10.1038/nature05284

[21] Dan J, Liu Y, Liu N, Chiourea M, Okuka M, Wu T, et al. (2014). Rif1 Maintains Telomere Length Homeostasis of ESCs by Mediating Heterochromatin Silencing. Dev Cell, 29(1):7-19. 24735877 10.1016/j.devcel.2014.03.004 PMC4720134

[22] Mei Y, Peng C, Liu Y Bin, Wang J, Zhou HH (2017). Silencing RIF1 decreases cell growth, migration and increases cisplatin sensitivity of human cervical cancer cells. Oncotarget, 8(63):107044-107051. 29291010 10.18632/oncotarget.22315 PMC5739795

[23] Wang H, Zhao A, Chen L, Zhong X, Liao J, Gao M, et al. (2009). Human RIF1 encodes an anti-apoptotic factor required for DNA repair. Carcinogenesis, 30(8):1314-1319. 19483192 10.1093/carcin/bgp136 PMC2718077

[24] Karaviti E, Kontogiannis A, Anastopoulos A, Kotteas E, Gomatou G (2023). An overview of the role of telomeres and telomerase in pre neoplastic lesions (Review). Mol Clin Oncol, 19(2):1-11. 10.3892/mco.2023.2657 PMC10326563 37424625

[25] Pirker C, Holzmann K, Spiegl-Kreinecker S, Elbling L, Thallinger C, Pehamberger H, et al. (2003). Chromosomal imbalances in primary and metastatic melanomas: over-representation of essential telomerase genes. Melanoma Res, 13(5):483-492. 14512790 10.1097/00008390-200310000-00007

[26] Wu KJ, Grandori C, Amacker M, Simon-Vermot N, Polack A, Lingner J, et al. (1999). Direct activation of TERT transcription by c-MYC. Nat Genet, 21(2):220-224. 9988278 10.1038/6010

[27] Castelo-Branco P, Choufani S, Mack S, Gallagher D, Zhang C, Lipman T, et al. (2013). Methylation of the TERT promoter and risk stratification of childhood brain tumours: An integrative genomic and molecular study. Lancet Oncol, 14(6):534-542. 23598174 10.1016/S1470-2045(13)70110-4

[28] Heaphy CM, Subhawong AP, Hong SM, Goggins MG, Montgomery EA, Gabrielson E, et al. (2011). Prevalence of the Alternative Lengthening of Telomeres Telomere Maintenance Mechanism in Human Cancer Subtypes. Am J Pathol, 179(4):1608-1615 21888887 10.1016/j.ajpath.2011.06.018 PMC3181356

[29] Henson JD, Neumann AA, Yeager TR, Reddel RR (2002). Alternative lengthening of telomeres in mammalian cells. Oncogene, 21(4):598-610. 11850785 10.1038/sj.onc.1205058

[30] Wu RC, Ayhan A, Maeda D, Kim KR, Clarke BA, Shaw P, et al. (2014). Frequent somatic mutations of the telomerase reverse transcriptase promoter in ovarian clear cell carcinoma but not in other major types of gynaecological malignancy. J Pathol, 232(4):473-481 24338723 10.1002/path.4315 PMC3946218

[31] Amin A, Morello M, Petrara MR, Rizzo B, Argenton F, De Rossi A, et al. (2023). Short-Term TERT Inhibition Impairs Cellular Proliferation via a Telomere Length-Independent Mechanism and Can Be Exploited as a Potential Anticancer Approach. Cancers, 15(10):2673. 37345011 10.3390/cancers15102673 PMC10216832

[32] Horn S, Figl A, Rachakonda PS, Fischer C, Sucker A, Gast A, et al. (1979). TERT promoter mutations in familial and sporadic melanoma. Science, 339(6122):959-961. 10.1126/science.1230062 23348503

[33] Huang FW, Hodis E, Xu MJ, Kryukov G V., Chin L, Garraway LA (1979). Highly recurrent TERT promoter mutations in human melanoma. Science, 339(6122):957-959. 10.1126/science.1229259 PMC4423787 23348506

[34] Ayhan A, Mao TL, Seckin T, Wu CH, Guan B, Ogawa H, et al. (2012). Loss of ARID1A Expression Is an Early Molecular Event in Tumor Progression From Ovarian Endometriotic Cyst to Clear Cell and Endometrioid Carcinoma. Int J Gynecol Cancer, 22(8):1310-1315. 22976498 10.1097/IGC.0b013e31826b5dcc PMC3460070

[35] Yamamoto S, Tsuda H, Takano M, Tamai S, Matsubara O (2012). Loss of ARID1A protein expression occurs as an early event in ovarian clear-cell carcinoma development and frequently coexists with PIK3CA mutations. Mod Pathol, 25(4):615-24. 22157930 10.1038/modpathol.2011.189

[36] Wiegand KC, Shah SP, Al-Agha OM, Zhao Y, Tse K, Zeng T, et al. (2010). ARID1A Mutations in Endometriosis-Associated Ovarian Carcinomas. N Engl J Med, 363(16):1532-43. 20942669 10.1056/NEJMoa1008433 PMC2976679

[37] Maeda D, Mao TL, Fukayama M, Nakagawa S, Yano T, Taketani Y, et al (2010). Clinicopathological Significance of Loss of ARID1A Immunoreactivity in Ovarian Clear Cell Carcinoma. Int J Mol Sci, 11(12):5120-5128. 21614196 10.3390/ijms11125120 PMC3100854

[38] Campbell PJ (2012). Telomeres and cancer: From crisis to stability to crisis to stability. Cell, 148(4):633-635. 22341437 10.1016/j.cell.2012.01.043 PMC3322332

[39] Killela PJ, Reitman ZJ, Jiao Y, Bettegowda C, Agrawal N, Diaz LA, et al. (2013). TERT promoter mutations occur frequently in gliomas and a subset of tumors derived from cells with low rates of self-renewal. Proc Natl Acad Sci, 110(15):6021-6026. 23530248 10.1073/pnas.1303607110 PMC3625331

[40] Ayhan A, Kuhn E, Wu RC, Ogawa H, Bahadirli-Talbott A, Mao TL, et al. (2017). CCNE1 copy-number gain and overexpression identify ovarian clear cell carcinoma with a poor prognosis. Mod Pathol, 30(2):297-303. 27767100 10.1038/modpathol.2016.160

[41] Huang HN, Chiang YC, Cheng WF, Chen CA, Lin MC, Kuo KT (2015). Molecular alterations in endometrial and ovarian clear cell carcinomas: clinical impacts of telomerase reverse transcriptase promoter mutation. Mod Pathol, 28(2):303-11. 25081752 10.1038/modpathol.2014.93

[42] Xue Y, Marvin ME, Ivanova IG, Lydall D, Louis EJ, Maringele L. Rif1 and Exo1 regulate the genomic instability following telomere losses (2016). Aging Cell, 15(3):553-562. 27004475 10.1111/acel.12466 PMC4854909

[43] Liu Y bin, Mei Y, Long J, Zhang Y, Hu DL, Zhou HH (2022). RIF1 promotes human epithelial ovarian cancer growth and progression via activating human telomerase reverse transcriptase expression. J Exp Clin Cancer Res, 37(1):1-15. 10.1186/s13046-018-0854-8 PMC6091081 30075819

[44] Liu Y bin, Mei Y, Tian ZW, Long J, Luo CH, Zhou HH (2022). Downregulation of RIF1 Enhances Sensitivity to Platinum-Based Chemotherapy in Epithelial Ovarian Cancer (EOC) by Regulating Nucleotide Excision Repair (NER) Pathway. Cell Physiol Biochem, 46(5):1971-84. 10.1159/000489418 29719287

[45] Mei Y, Liu Y bin, Cao S, Tian ZW, Zhou HH (2018). RIF1 promotes tumor growth and cancer stem cell-like traits in NSCLC by protein phosphatase 1-mediated activation of Wnt/β-catenin signaling. Cell Death Dis, 9(10):1-17. 30237512 10.1038/s41419-018-0972-4 PMC6148239

[46] Mattarocci S, Hafner L, Lezaja A, Shyian M, Shore D (2016). Rif1: A conserved regulator of DNA replication and repair hijacked by telomeres in yeasts. Front Genet, 7-45. 27066066 10.3389/fgene.2016.00045 PMC4811881

[47] Mattarocci S, Reinert J, Bunker RD, Theraputics MR, Fontana GA, Zurich E (2017). Rif1 maintains telomeres and mediates DNA repair by encasing DNA ends. Nat Struct Mol Biol, 24:588-595. 28604726 10.1038/nsmb.3420

[48] Kanoh J, Ishikawa F (2001). spRap1 and spRif1, recruited to telomeres by Taz1, are essential for telomere function in fission yeast. Curr Biol, (20):1624-30. 11676925 10.1016/s0960-9822(01)00503-6

[49] Malyavko A, Petrova O, Zvereva M (2019). Functional duplication of Rap1 in methylotrophic yeasts. Sci Rep, 9:7196. 31076582 10.1038/s41598-019-43595-8 PMC6510891

[50] Malyavko A, Naturae ODA (2020). The telomeric Cdc13 protein from yeast Hansenula polymorpha. Acta Naturae, 12(1):84-88. 32477602 10.32607/actanaturae.10944 PMC7245964

[51] Malyavko AN, Petrova OA, Zvereva MI, Polshakov VI, Dontsova OA (2022). Telomere length regulation by Rif1 protein from Hansenula polymorpha. Elife, 1;11. 10.7554/eLife.75010 PMC8820739 35129114

[52] Moriyama K, Yoshizawa-Sugata N, Masai H (2018). Oligomer formation and G-quadruplex binding by purified murine Rif1 protein, a key organizer of higher-order chromatin architecture. J Biol Chem, 293(10):3607-3624. 29348174 10.1074/jbc.RA117.000446 PMC5846147

[53] Saha D, Singh A, Hussain T, Srivastava V, Sengupta S, Kar A, et al. (2017). Epigenetic suppression of human telomerase (hTERT) is mediated by the metastasis suppressor NME2 in a G-quadruplex- dependent fashion. J Biol Chem, 292(37):15205-15215. 28717007 10.1074/jbc.M117.792077 PMC5602382

[54] Rice C, Skordalakes E (2016). Structure and function of the telomeric CST complex. Comput Struct Biotechnol J. 14:161-7. 27239262 10.1016/j.csbj.2016.04.002 PMC4872678

[55] Mason JM, Frydrychova RC, Biessmann H (2008). Drosophila telomeres: An exception providing new insights. BioEssays, 30(1):25-37. 18081009 10.1002/bies.20688 PMC2804870

[56] Seller CA, O’Farrell PH (2018). Rif1 prolongs the embryonic S phase at the Drosophila mid-blastula transition. PLoS Biol, 16(5):e2005687. 29746464 10.1371/journal.pbio.2005687 PMC5963817

[57] Cho CY, Seller CA, O’Farrell PH (2022). Temporal control of late replication and coordination of origin firing by self-stabilizing Rif1-PP1 hubs in Drosophila. Proc Natl Acad Sci, 119(26): e2200780119. 35733247 10.1073/pnas.2200780119 PMC9245680

[58] Sreesankar E, Senthilkumar R, Bharathi V, Mishra RK, Mishra K (2012). Functional diversification of yeast telomere associated protein, Rif1, in higher eukaryotes. BMC Genom, 13:255. 10.1186/1471-2164-13-255 PMC3410773 22712556

[59] Ribeyre C (2012). Anticheckpoint pathways at telomeres in yeast. Nat Struct Mol Biol, 19:307-313. 22343724 10.1038/nsmb.2225

[60] Anbalagan S, Bonetti D, Lucchini G, Longhese MP (2011). Rif1 supports the function of the CST complex in yeast telomere capping. PLoS Genet, 7(3): e1002024. 21437267 10.1371/journal.pgen.1002024 PMC3060071

[61] Shubin CB, Greider CW (2020). The role of Rif1 in telomere length regulation is separable from its role in origin firing. Elife, 9:1-40. 10.7554/eLife.58066 PMC7371424 32597753

[62] Wang J, Zhang H, al Shibar M, Willard B, Ray A, Runge KW (2018). Rif1 phosphorylation site analysis in telomere length regulation and the response to damaged telomeres. DNA Repair, 65:26-33. 29544213 10.1016/j.dnarep.2018.03.001 PMC5911405

[63] Sholes SL, Karimian K, Gershman A, Kelly TJ, Timp W, Greider CW (2022). Chromosome-specific telomere lengths and the minimal functional telomere revealed by nanopore sequencing. Genome Res, 32(4):616-28. 34702734 10.1101/gr.275868.121 PMC8997346

[64] Kedziora S, Gali VK, Wilson RHC, Clark KRM, Nieduszynski CA, Hiraga SI, et al. (2018). Rif1 acts through Protein Phosphatase 1 but independent of replication timing to suppress telomere extension in budding yeast. Nucleic Acids Res, 46(8):3993-4003. 29529242 10.1093/nar/gky132 PMC5934629

[65] di Virgilio M, Callen E, Yamane A, Zhang W, Jankovic M, Gitlin AD, et al. (1979). Rif1 prevents resection of DNA breaks and promotes immunoglobulin class switching. Science, 339(6120):711-715. 10.1126/science.1230624 PMC3815530 23306439

[66] Fontana GA, Hess D, Reinert JK, Mattarocci S, Falquet B, Klein D, et al (2019). Rif1 S-acylation mediates DNA double-strand break repair at the inner nuclear membrane. Nat Commun, 10(1):1-14. 31182712 10.1038/s41467-019-10349-z PMC6557901

[67] Fontana GA, Reinert JK, Thomä NH, Rass U (2018). Shepherding DNA ends: Rif1 protects telomeres and chromosome breaks. Microb Cell, 5(7):327-343. 29992129 10.15698/mic2018.07.639 PMC6035837

[68] Rosas-Hernández LL, Juárez-Reyes A, Arroyo-Helguera OE, de Las Peñas A, Pan SJ, Cormack BP, et al. (2008). yKu70/yKu80 and Rif1 regulate silencing differentially at telomeres in Candida glabrata. Eukaryot Cell, 7(12):2168-2178. 18836091 10.1128/EC.00228-08 PMC2593182

[69] Amano T, Hirata T, Falco G (2013). Zscan4 restores the developmental potency of embryonic stem cells. Nat Commun, 4:1966. 23739662 10.1038/ncomms2966 PMC3682791

[70] Dan J, Zhou Z, Wang F, Wang H, Guo R, Keefe DL, et al. (2022). Zscan4 Contributes to Telomere Maintenance in Telomerase-Deficient Late Generation Mouse ESCs and Human ALT Cancer Cells. Cells, 11(3):456. 35159266 10.3390/cells11030456 PMC8834411

[71] Zalzman M, Falco G, Sharova L (2010). Zscan4 regulates telomere elongation and genomic stability in ES cells. Nature, 464:858-863. 20336070 10.1038/nature08882 PMC2851843

[72] Peace JM, Ter-Zakarian A, Aparicio OM (2014). Rif1 regulates initiation timing of late replication origins throughout the S. cerevisiae genome. PLoS One, 9(5): e98501. 24879017 10.1371/journal.pone.0098501 PMC4039536

[73] Zimmermann M, Lottersberger F, Buonomo SB, Sfeir A, de Lange T (2013). 53BP1 regulates DSB repair using Rif1 to control 5′ end resection. Science, 339(6120):700-704. 23306437 10.1126/science.1231573 PMC3664841

[74] Buonomo S, Wu Y, Ferguson D (2009). Mammalian Rif1 contributes to replication stress survival and homology-directed repair. J Cell Biol, 187(3):385-398. 19948482 10.1083/jcb.200902039 PMC2779251

[75] Shweiki D, Itin A, Soffer D, Keshet E (1992). Vascular endothelial growth factor induced by hypoxia may mediate hypoxia-initiated angiogenesis. Nature, 359(6398):843-845. 1279431 10.1038/359843a0

[76] Pugh CW, Ratcliffe PJ (2017). New horizons in hypoxia signaling pathways. Exp Cell Res, 356(2):116-121. 28315322 10.1016/j.yexcr.2017.03.008 PMC5653532

[77] Corbet C, Feron O (2017). Tumour acidosis: from the passenger to the driver’s seat. Nat Rev Cancer, 17(10):577-593. 28912578 10.1038/nrc.2017.77

[78] Kondo A, Yamamoto S, Nakaki R, Shimamura T, Hamakubo T, Sakai J, et al (2017). Extracellular Acidic pH Activates the Sterol Regulatory Element-Binding Protein 2 to Promote Tumor Progression. Cell Rep, 18(9):2228-2242. 28249167 10.1016/j.celrep.2017.02.006

[79] Dey P, Kimmelman A (2021). Metabolic Codependencies in the Tumor Microenvironment. Am Assoc Cancer Discov, 11(5):1067-1081. 10.1158/2159-8290.CD-20-1211 PMC8102306 33504580

[80] Yang L, Achreja A, Yeung TL, Mangala LS, Jiang D, Han C, et al. (2016). Targeting Stromal Glutamine Synthetase in Tumors Disrupts Tumor Microenvironment-Regulated Cancer Cell Growth. Cell Metab, 24(5):685-700. 27829138 10.1016/j.cmet.2016.10.011 PMC7329194

[81] Mukherjee A, Chiang CY, Daifotis HA, Nieman KM, Fahrmann JF, Lastra RR, et al. (2020). Adipocyte-induced FABP4 expression in ovarian cancer cells promotes metastasis and mediates carboplatin resistance. Cancer Res, 80(8):1748-1761. 32054768 10.1158/0008-5472.CAN-19-1999 PMC10656748

[82] Mockler MB, Conroy MJ, Lysaght J (2014). Targeting T cell immunometabolism for cancer immunotherapy; understanding the impact of the tumor microenvironment. Front Oncol, 4:107. 24904823 10.3389/fonc.2014.00107 PMC4032940

[83] Shen L, Xia M, Zhang Y, Luo H, Dong D, Sun L (2021). Mitochondrial integration and ovarian cancer chemotherapy resistance. Exp Cell Res, 401(2):112549. 33640393 10.1016/j.yexcr.2021.112549

[84] Wei X, Li X, Yan W, Zhang X, Sun Y, Zhang F (2018). SKP2 Promotes Hepatocellular Carcinoma Progression Through Nuclear AMPK-SKP2-CARM1 Signaling Transcriptionally Regulating Nutrient-Deprived Autophagy Induction. Cell Physiol Biochem, 47(6):2484-2497. 29991055 10.1159/000491622

[85] Mijit M, Caracciolo V, Melillo A, Amicarelli F, Giordano A (2020). Role of p53 in the Regulation of Cellular Senescence. Biomolecules, 10(3):420. 32182711 10.3390/biom10030420 PMC7175209

[86] Yang M, Li J, Gu P, Fan X (2021). The application of nanoparticles in cancer immunotherapy: Targeting tumor microenvironment. Bioact Mater, 6(7):1973-1987. 33426371 10.1016/j.bioactmat.2020.12.010 PMC7773537

[87] Kwon Y, Smith B, Zhou Y, Kaufman M (2015). Effective inhibition of c-MET-mediated signaling, growth and migration of ovarian cancer cells is influenced by the ovarian tissue microenvironment. Oncogene, 34:144-153. 24362531 10.1038/onc.2013.539 PMC4067476

[88] Yamamoto S, Tsuda H, Takano M, Tamai S, Matsubara O (2012). PIK3CA mutations and loss of ARID1A protein expression are early events in the development of cystic ovarian clear cell adenocarcinoma. Virchows Archiv, 460(1):77-87. 22120431 10.1007/s00428-011-1169-8

[89] Rahman MT, Nakayama K, Rahman M, Nakayama N, Ishikawa M, Katagiri A, et al. (2012). Prognostic and therapeutic impact of the chromosome 20q13.2 ZNF217 locus amplification in ovarian clear cell carcinoma. Cancer, 118(11):2846-57. 22139760 10.1002/cncr.26598

[90] Ho CM, Lin MC, Huang SH, Huang CJ, Lai HC, Chien TY, et al. (2009). PTEN promoter methylation and LOH of 10q22-23 locus in PTEN expression of ovarian clear cell adenocarcinomas. Gynecol Oncol, 112(2):307-313. 19007975 10.1016/j.ygyno.2008.09.040

[91] Rahman M, Nakayama K, Rahman MT, Nakayama N, Ishikawa M, Katagiri A, et al. (2012). Clinicopathologic and biological analysis of PIK3CA mutation in ovarian clear cell carcinoma. Hum Pathol, 43(12):2197-206. 22705003 10.1016/j.humpath.2012.03.011

